# Thymoma with Intravascular Tumor Thrombus in the Left Brachiocephalic Vein: A Case Report

**DOI:** 10.70352/scrj.cr.25-0118

**Published:** 2025-07-01

**Authors:** Taimei Tachibana, Yosuke Matsuura, Hironori Ninomiya, Yoshinao Sato, Ayumi Suzuki, Junji Ichinose, Masayuki Nakao, Sakae Okumura, Norihiko Ikeda, Mingyon Mun

**Affiliations:** 1 Department of Thoracic Surgical Oncology, Cancer Institute Hospital, Japanese Foundation for Cancer Research, Tokyo, Japan; 2 Department of Surgery, Tokyo Medical University, Tokyo, Japan; 3 Division of Pathology, Cancer Institute, Japanese Foundation for Cancer Research, Tokyo, Japan; 4 Department of Pathology, Cancer Institute Hospital, Japanese Foundation for Cancer Research, Tokyo, Japan; 5 Department of Diagnostic Imaging Center, Cancer Institute Hospital, Japanese Foundation for Cancer Research, Tokyo, Japan

**Keywords:** thymoma, tumor thrombus, vascular invasion, intravascular growth

## Abstract

**INTRODUCTION:**

Thymomas have the potential to locally invade and metastasize, occasionally infiltrating adjacent structures, such as the great vessels and the heart. Although direct extension is the primary mechanism of vascular invasion, rare cases of intravascular growth have also been reported.

**CASE PRESENTATION:**

We present the case of a 50-year-old woman diagnosed with a thymoma that extended intraluminally into the left brachiocephalic vein (LBCV), forming a tumor thrombus. The patient was referred to our hospital after chest computed tomography (CT), which revealed an anterior mediastinal tumor with a filling defect adjacent to the superior aspect of the tumor. Initially, the defect was thought to be a blood clot because of the preserved vascular wall structure. However, follow-up CT scans conducted 2 weeks later revealed persistence of the defect and a slight increase in size, leading to the diagnosis of a tumor thrombus. Further imaging, including contrast-enhanced CT and magnetic resonance imaging, confirmed thymoma invasion of the LBCV, necessitating surgical intervention. The patient underwent a median sternotomy and tumor resection with combined partial resection of the LBCV and right upper lobe. Intraoperatively, a dilated thymic vein continuous with the tumor was identified. The tumor thrombus was visible through the LBCV wall, aiding in the determination of its extent. The LBCV was clamped proximally and distally, and the dilated thymic vein was ligated and divided. Subsequently, thymectomy encompassing the tumor and partial resection of the LBCV wall were performed to remove the thrombus. Microscopically, the tumor was classified as a type B2 thymoma. No evidence of continuity between the tumor thrombus and the thymic vein was observed. No postoperative complication was observed. Nine months after surgery, the patient experienced recurrence with pleural dissemination and underwent resection.

**CONCLUSIONS:**

Thymomas can invade vessels through intravascular growth, and contrast-enhanced CT is important for accurately diagnosing such cases. In this instance, preoperative identification of the tumor thrombus enabled a comprehensive surgical approach, resulting in complete resection of the tumor and thrombus, without the need for embolization. This case underscores the significance of meticulous imaging and surgical planning in the management of complex thymomas to ensure optimal patient outcomes.

## Abbreviations

CT
: computed tomography
FDG
: fluorodeoxyglucose
LBCV
: left brachiocephalic vein
RA
: right atrium
SVC
: superior vena cava

## INTRODUCTION

Thymomas are malignant neoplasms originating from the epithelial components of the thymus.^1)^ Thymic epithelial tumors are the most prevalent primary malignant tumors of the mediastinum, with an incidence rate ranging from 0.44 to 0.68 cases per 100000 individuals. When complete resection is feasible, surgical resection is considered the standard treatment for thymic epithelial tumors. Conversely, in cases of incomplete resection (R1 or R2), postoperative radiotherapy or chemoradiotherapy is recommended to enhance local control and mitigate the risk of recurrence.^2)^ Thymic epithelial tumors have the potential for local invasion and metastasis, with occasional infiltration of adjacent structures, such as the great vessels and the heart. Direct extension is the predominant mechanism underlying vascular invasion. Although rare, intravascular growth has been documented in select cases.^3)^ Herein, we present a rare case of thymoma with an intravascular tumor thrombus floating within the lumen of the left brachiocephalic vein (LBCV) via the thymic vein.

## CASE PRESENTATION

**Fig. 1:**
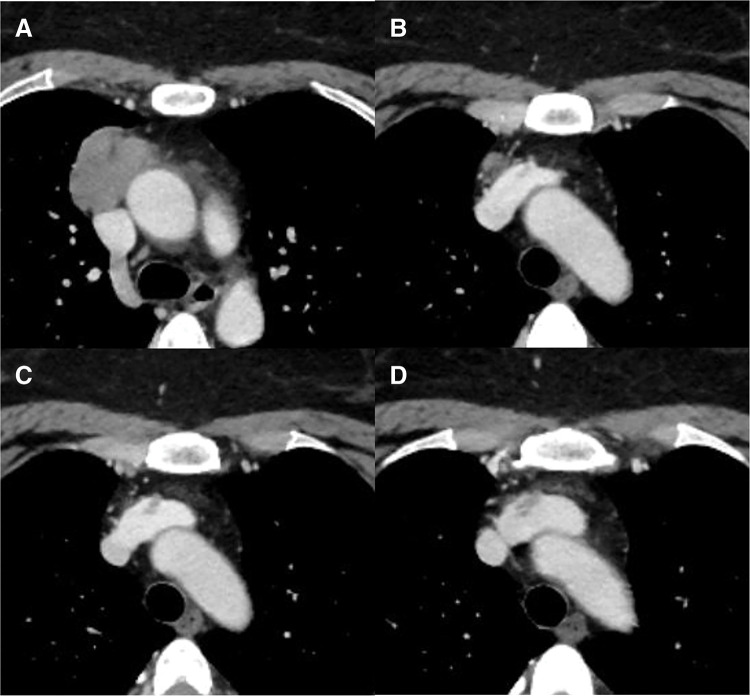
Preoperative imaging of the lesion. (**A**) Chest computed tomography (CT) displaying a mass (6.3 × 4.1 × 3.1 cm) in the anterior mediastinum. (**B**) The superior margin of the tumor. (**C**) A filling defect was observed in the left brachiocephalic vein. (**D**) A filling defect was also observed in the left brachiocephalic vein on follow-up CT.

**Fig. 2:**
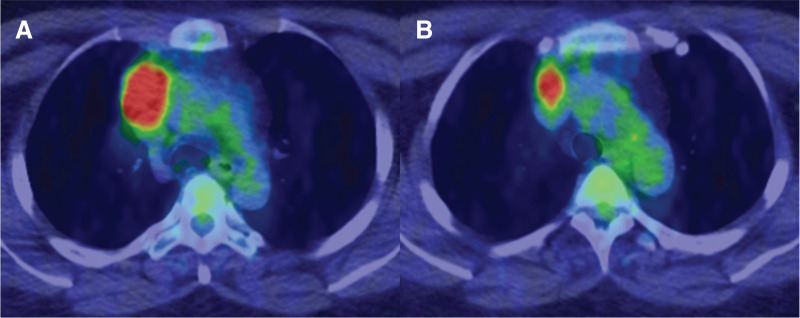
Preoperative FDG-PET/CT findings. (**A**) FDG-PET/CT scan shows a maximum standardized uptake value of 10.39 in the main tumor mass. (**B**) A strong FDG uptake was observed up to the height of the tumor thrombus. CT, computed tomography; FDG, fluorodeoxyglucose; PET, positron emission tomography

A 50-year-old woman was referred to our hospital after chest computed tomography (CT) for chest pain, which revealed an anterior mediastinal tumor. The patient had no relevant medical history. Chest CT revealed a 6.3 × 4.1 × 3.1 cm tumor in the anterior mediastinum (**Fig. 1A**). A filling defect was observed in the LBCV adjacent to the superior aspect of the tumor (**Fig. 1B**), which extended cranially (**Fig. 1C**). The filling defect was barely continuous from the cranial side of the tumor, and the vessel wall structure was preserved, as observed on the CT scan. However, on the follow-up CT scan, obtained 2 weeks later, the filling defect remained at the same location with a slight increase in size (**Fig. 1D**). Furthermore, fluorodeoxyglucose (FDG)-positron emission tomography/CT showed a maximum standardized uptake value of 10.39 in the main tumor mass (**Fig. 2A**), and strong FDG uptake was observed up to the height of the tumor thrombus (**Fig. 2B**). Additionally, D-dimer level was not elevated at 0.5 μg/mL. Based on these findings, the filling defect was determined to be a tumor thrombus. The patient was diagnosed with invasive thymoma, but there were no symptoms of myasthenia gravis, and the serum anti-acetylcholine receptor antibody level was within the normal range.

**Fig. 3:**
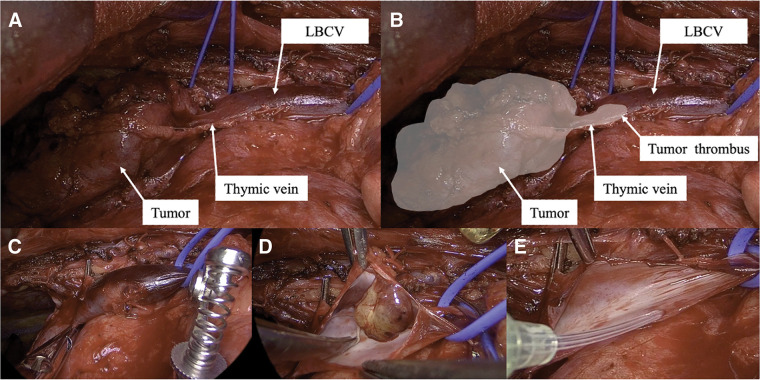
Operative findings. (**A**) The tumor was attached to the left brachiocephalic vein, and dissection revealed a dilated thymic vein. (**B**) The area outlined in white indicates the extent of the tumor, including the tumor thrombus. It suggests intravascular extension via the thymic vein rather than direct invasion. (**C**) After clamping the proximal and distal portions of the left brachiocephalic vein containing the tumor thrombus, the dilated thymic vein was ligated and then divided. (**D**) Tumor thrombus in the left brachiocephalic vein. (**E**) The vascular wall was resected and the tumor thrombus removed. LBCV, left brachiocephalic vein

**Fig. 4:**
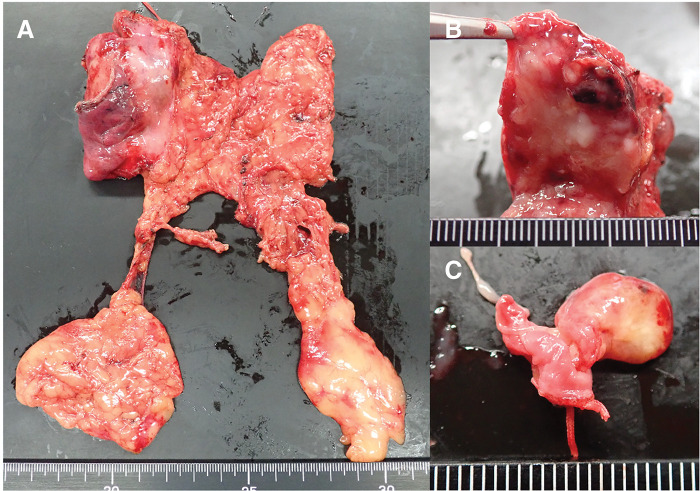
Excised specimen. (**A**) The tumor measured 4.5 × 4.2 × 2.9 cm and was located within the thymus. (**B**) The cut surface was firm and showed multiple lobules bordered by white fibrous septa. (**C**) There was a tumor thrombus of 1.0 cm.

**Fig. 5:**
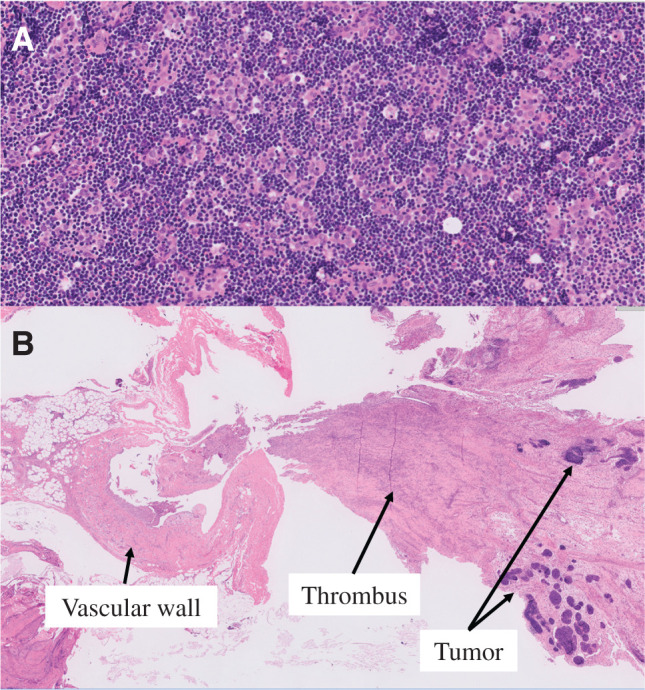
Histological findings. (**A**) Clusters of epithelioid cells were observed among abundant lymphocytes, leading to the diagnosis of type B2 thymoma. (**B**) Clusters of tumor cells scattered in the thrombus. No direct connection between the tumor thrombus and the thymic vein was observed.

The patient underwent a median sternotomy and tumor resection with combined partial resection of the LBCV and right upper lobe (**Video**). The left side of the thymus was dissected, and the periphery of the LBCV was taped with a vessel loop. The tumor was adherent to the right lung. Because of the tumor, it was not possible to establish the area of the confluence of the LBCV and superior vena cava (SVC). The right brachiocephalic vein was taped with a vessel loop. The thymic vein and surrounding tissue were connected to the tumor (**Fig. 3A**). A tumor embolus that moved with the pulsation of blood flow could be seen through the vessel wall within the LBCV, and the extent of the tumor embolus could be identified. The tumor was identified as a white area (**Fig. 3B**). The central part of the thymic vein was clamped with vascular forceps, and the tumor side was separated. This maneuver allowed the tumor to move into the right thoracic cavity, and we were able to confirm the confluence of the LBCV and SVC. After removing the thymus gland, including the main tumor mass, we began to remove the tumor embolus. We clamped the central and peripheral portions of the LBCV with vascular clamping forceps (**Fig. 3C**). As the vein was clamped for only a short time, heparin was not administered. When the vessel wall of the LBCV was incised, the tumor emboli were found to be clearly separated from the surrounding vessel wall (**Fig. 3D**). As only few areas of vessel wall defect were noted (**Fig. 3E**), they were closed directly with sutures and covered with Tacho-Seal. The tumor measured 4.5 × 4.2 × 2.9 cm and was located within the thymus (**Fig. 4A**). The cut surface was firm and contained multiple lobules bordered by white fibrous septa (**Fig. 4B**). In addition, a 1.0 cm tumor thrombus with its base in the LBCV was identified (**Fig. 4C**). Microscopically, clusters of epithelioid cells were observed among the abundant lymphocytes, leading to a diagnosis of type B2 thymoma (**Fig. 5A**). Neither hematoxylin and eosin nor Elastica van Gieson staining showed continuity between the tumor thrombus and the thymic vein (**Fig. 5B**).

No postoperative complications were observed. Post-operative follow-up was performed without adjuvant chemotherapy or radiotherapy. Nine months after surgery, the patient experienced recurrence with pleural dissemination and underwent resection of the tumor deposits in the pleura, along with partial pulmonary resection.

## DISCUSSION

Invasion of the major vessels, particularly the LBCV and superior vena cava, is relatively common in advanced thymomas. However, most of these cases involve direct invasion of the vessels by the tumor. This rare case presented here demonstrates tumor invasion into the vascular lumen rather than direct infiltration of major vessels. It has been hypothesized that thymomas enter great vessels through small tributaries, such as the thymic veins, or via focal transmural invasion, leading to intraluminal tumor growth along the venous system, similar to the angioinvasive behavior observed in renal cell carcinoma. This mode of spread, which involves minimal or no destruction of the vessel wall, is distinct from direct invasion in which the tumor aggressively penetrates the vascular adventitia and media.

Preoperative contrast studies are critical not only for surgical planning but also to improve the assessment of vascular invasion of the tumor. We conducted a focused literature search using the PubMed database for studies published up to April 2025, using the keywords “thymoma” and “intravascular.” We further filtered for surgically treated cases and summarized them in **Table 1**. In most previously reported cases, the tumor thrombi were large and clearly demonstrated intravascular growth on preoperative imaging.^4–11)^ Our case is rare because, on imaging, we detected intravascular invasion, characterized by linear visualization of the tumor within the LBCV. This diagnosis was facilitated by performing contrast-enhanced CT twice preoperatively, which allowed accurate detection of the intravascular invasion.

**Table 1:** Summary of previous reports on surgical resection of thymomas with intravascular grow

Year	Authors	Age	Sex	Clinical presentation	Main tumor size (cm)	Tumor thrombus size (cm)	Infiltrating blood vessel	Surgical procedure
1996	Sato et al.^4)^	43	F	Facial swelling	N.D.	N.D.	LBCV, SVC, RA	Graft reconstruction
1999	Nomori et al.^5)^	70	M	Eyelid ptosis (myasthenia gravis)	5.2	N.D.	LBCV	N.D.
2010	Li et al.^6)^	40	N.D.	SVC syndrome and chest tightness	6.5	7.0	LBCV, SVC, RA	Graft reconstruction
2012	Toker et al.^7)^	53	F	SVC syndrome	8.0	3.9	SVC, RA	Graft reconstruction
2013	Rizzardi et al.^8)^	44	F	Facial and left upper limb edema	5.0	N.D.	LBCV, SVC, RA	Direct suture
2013	Kim et al.^9)^	39	F	SVC syndrome	N.D.	N.D.	LBCV, SVC, RA	N.D.
2018	Kawakita et al.^10)^	84	M	Incidental abnormal chest radiographic findings	4.4	N.D.	LBCV	Direct suture
2020	Shen et al.^11)^	63	F	Dyspnea, and upper limb and facial edema	12.0	N.D.	LBCV, SVC, RA	Graft reconstruction
2025	Our case	50	F	Chest pain	4.5	1.0	LBCV	Direct suture

F, female; LBCV, left brachiocephalic vein; M, male; N.D., not documented; RA, right atrium; SVC, superior vena cava

One previous report described a case involving intravascularly invasive thymomas in which intraoperative tumor embolization resulted in pulmonary artery obstruction, necessitating reoperation for pulmonary embolectomy.^12)^ By contrast, in our case, tumor embolization was successfully prevented by clamping both the central and peripheral portions of the LBCV with vascular clamps and ligating the thymic vein. Although a tumor thrombus was visible in this case, ultrasound imaging may be useful in more challenging scenarios to identify and delineate the extent of intravascular tumor invasion intraoperatively.

## CONCLUSIONS

We reported a rare case of thymoma with vascular invasion due to intravascular growth. Our findings indicate that preoperative contrast-enhanced CT plays a crucial role in identifying tumor thrombi. By clamping the affected vessel and ligating the thymic vein, we successfully resected the tumor thrombus along with the tumor itself, without causing embolization. This case underscores the significance of meticulous imaging and surgical planning in the management of complex thymomas to ensure optimal patient outcomes.

## DECLARATIONS

### Funding

None.

### Authors’ contributions

TT and YM wrote this paper.

All authors read and approved the final manuscript.

### Availability of data and materials

Not applicable.

### Ethics approval and consent to participate

Consent was obtained from the patient for all procedures, and this case report was approved by the Institutional Review Board of Cancer Institute Hospital (Approval Number: 2023-GB-078).

### Consent for publication

Written informed consent was obtained from the patient for the use of her data for research and publication of this case report.

### Competing interests

The authors have no competing interests to declare.

## SUPPLEMENTARY MATERIALS

Supplementary Video
